# Increasing Interpreter Use for Pediatric Cardiology Units: A Quality Improvement Initiative

**DOI:** 10.1097/pq9.0000000000000933

**Published:** 2026-09-28

**Authors:** Dulce Maria E. Ovalle Sanchez, Jennifer Gauntt, Ronna Porterfield, Robert J. Gajarski

**Affiliations:** From the * The Heart Center at Nationwide Children’s Hospital, Columbus, Ohio; † Center for Clinical Excellence at Nationwide Children’s Hospital, Columbus, Ohio; ‡ Heart Center at Rocky Mountain Children’s Hospital, Denver, Colo.

## Abstract

**Introduction:**

Effective communication between healthcare teams, patients, and families is critical to achieving high-quality care. Despite US regulations requiring language services, interpreters remain underutilized, and there is no standardized approach to communication in hospital settings. This quality improvement project aimed to increase interpreter use for families who speak a language other than English (LOE) from 94 to 122 occurrences/100 LOE-patient-days, the average interpreter interaction time/patient/month from 10 to 13 minutes, and the average interpreter use percentage during emergencies from 29% to 44% by December 31, 2025, in the acute care cardiology and cardiothoracic settings.

**Methods:**

Baseline data from January 2022 to August 2024 were evaluated, and implemented interventions focused on 3 categories: increased interpreter accessibility, interpreter-use education, and the creation of translated documents. The number of interpreter occurrences and average interpreter minutes/patient/month, indexed to LOE-patient-days, were tracked monthly using statistical process control charts. A statistical process control p-chart was used to track the number of patients for whom an interpreter was used during emergencies as a function of total LOE patients who experienced an emergency with parents present.

**Results:**

Interpreter use increased from 94 to 212 occurrences/100 LOE-patient-days whereas the average interpreter minutes/patient/month increased from 10 to 23 minutes following the interventions. The percentage of interpreter use during emergencies increased from 29% to 91%. Process and balancing measures tracked intervention utilization and unintended consequences.

**Conclusions:**

Interventions that directly increased interpreter use helped standardize care for families who spoke an LOE, thereby enhancing healthcare communication, literacy, and family engagement.

## INTRODUCTION

Effective communication among healthcare teams, patients, and families is essential for high-quality care. Hospitalization is often stressful and medically complex, and these challenges may be amplified for families who speak a language other than English (LOE). Nearly 70 million people in the United States speak an LOE.^1^ Patients and families who speak an LOE experience discrepancies in healthcare comprehension,^2,3^ patient satisfaction,^4^ length of stay (LOS),^4–7^ adverse events,^4,8^ adherence,^2^ readmissions,^4,5,9^ and overall quality of care.^10,11^ Professional interpreter services improve communication and healthcare quality; however, interpreter use remains underutilized in the United States and internationally.^12–17^

Within our pediatric acute care cardiology unit (ACCU) and cardiothoracic intensive care unit (CTICU), internal review demonstrated similar underutilization of interpreter services among the approximately 15% of patients and families who spoke an LOE. The most common languages were Spanish, Somali, Nepali, Arabic, and Haitian Creole. To address this gap, we conducted a quality improvement (QI) initiative using Institute for Healthcare Improvement methodology. Our primary aim was to increase interpreter service occurrences for families who spoke an LOE by 30%, from 94 to 122 occurrences per 100 LOE-patient-days, by December 31, 2025. Secondary aims were to increase average interpreter minutes per patient per month, indexed to LOE patient days, by 30%, from 10 to 13 minutes, and increase interpreter use during emergencies by 50%, from 29% to 44%. Previous QI studies have improved interpreter use in pediatric intensive care units and emergency settings,^18–21^ but no standardized approach for inpatient pediatric hospitalizations exists yet. Our interventions aligned with institutional policy and federal requirements under Title VI of the Civil Rights Act and the Affordable Care Act mandating provision of language services at no cost to patients.^4,22–24^

## METHODS

### Context

This project is a single-center QI study conducted at our inpatient Heart Center within a freestanding, nonprofit quaternary-care pediatric hospital. The Heart Center comprises a 24-bed ACCU and a 20-bed CTICU. Annually, the ACCU and CTICU have roughly 1,500 and 750 admissions, respectively. Heart Center staff includes a multidisciplinary team of cardiologists, cardiac intensive care physicians, advanced practice nurse practitioners (APRNs), pediatric cardiology and pediatric critical care medicine fellows, pediatric residents, medical students, registered nurses (RNs), respiratory therapists, dietitians, social workers, and clinical pharmacists. The patient-to-nurse ratio varies by acuity, with averages of 3–4:1 and 1–2:1 in the ACCU and CTICU, respectively.

### Historical Practice

Historically, language services were accessed by calling the interpreter line to arrange an in-person interpreter, calling the interpreter line to speak by telephone, or initiating a conversation through an interpreter application downloaded onto individual electronic tablets by the institution. The available interpreters were either interpretation-certified hospital employees or trained interpreters through externally contracted interpreting agencies. Each inpatient unit was assigned 3 tablets on wheeled stands containing the predownloaded interpreter application, kept in the locked nurses’ medication room, accessible only with an RN badge. When a patient or family needed an interpreter, a nurse retrieved the tablet, entered its passcode, opened the interpreter application, and selected the language to start the conversation. The patient’s name, medical record number, and hospital unit were required to initiate the interpreter service. At the conclusion of the interpretation session, the nurse returned the tablet to the locked medication room.

The process for calling in-person and telephone interpreters was the same. To schedule in-person interpreters, healthcare providers called the interpreter line and asked to schedule an interpreter, providing the patient’s name, language, medical record number, and hospital unit. Similarly, to call an interpreter via telephone, healthcare providers called the interpreter line, requested the patient’s language, and then provided identical demographic data (see **Figure 1, Supplemental Digital Content 1,** https://links.lww.com/PQ9/A825).

### Preintervention

In 2023, the Heart Center spearheaded projects to improve language communication through multilingual discharge instructions, simulation projects, and family-centered rounds (FCRs). In July 2024, recognizing that our use of interpreter services was suboptimal, we assembled a multidisciplinary team to address the issue. The team consisted of ACCU and CTICU RNs, nursing managers, advanced practice nurse practitioners, cardiologists, cardiac intensivists, social workers, the director of language services, members of our institution’s Office of Health Equity, and a QI expert. We developed an anonymous survey for healthcare providers in August 2024 to understand perceived barriers to using interpreters and to assess providers’ self-reported frequency of using interpreters to communicate with patients and families. The survey was administered before the implementation of a series of interventions and readministered 1 year later. Additionally, we analyzed baseline ACCU and CTICU interpreter services report data from January 2022 to August 2024 provided by our language services department.

### Interventions

The project team met several times and, using standard QI tools, formulated a specific aim statement and designed a key driver diagram with a series of interventions based on the preintervention data (Fig. 1). The interventions were grouped into 3 categories: increasing accessibility of interpreter services, education on interpreter utilization, and creation of translated documents.

**Fig. 1.**
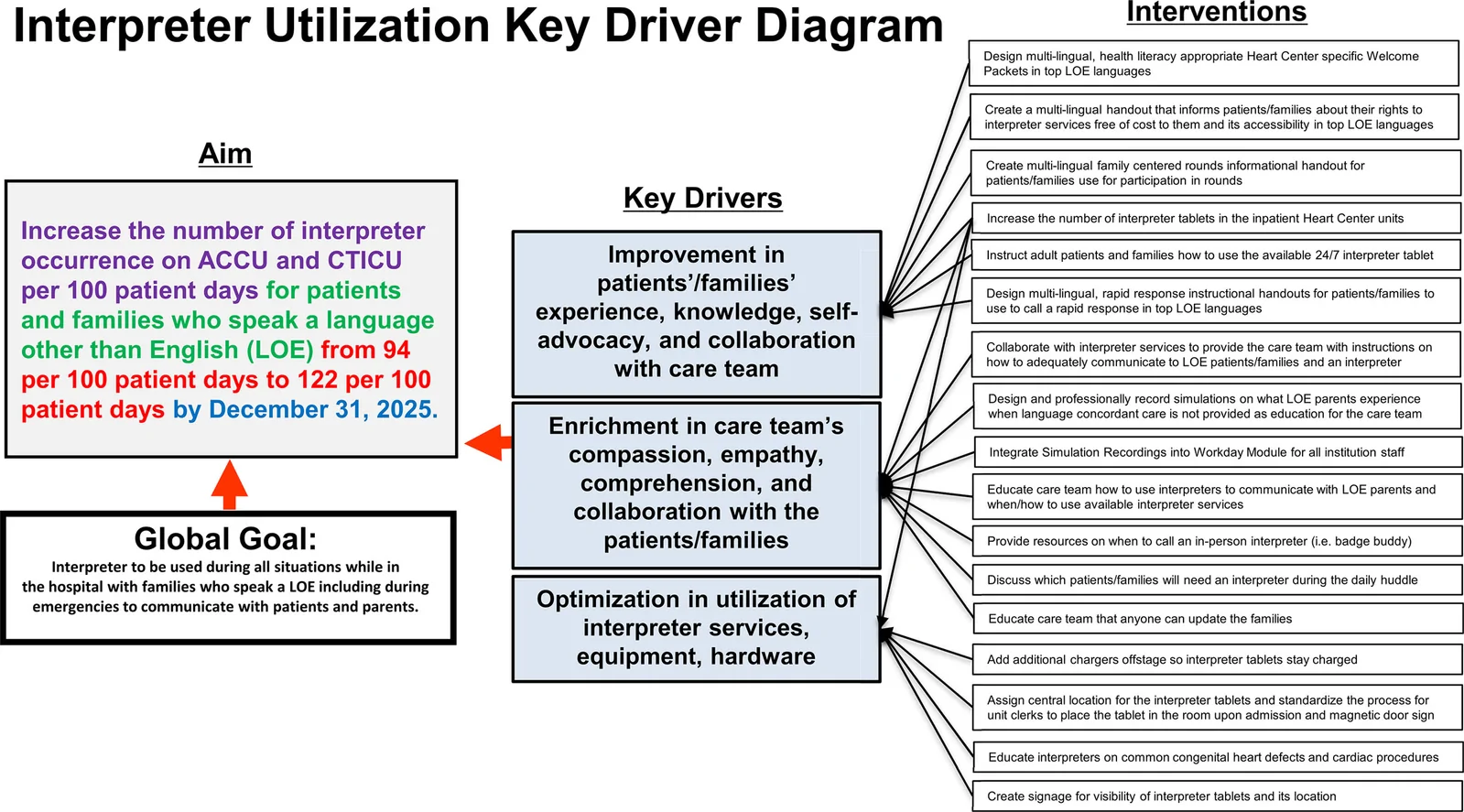
Key driver diagram.

### Accessibility of Interpreter Services

Before this initiative, 6 interpreter-dedicated tablets were available: 3 in the ACCU and 3 in the CTICU. In November 2024, using philanthropic donations, we increased the number of available tablets to 16, with 8 on each unit. We changed the storage location to a central area in the providers’ conference workspace where they would be easily accessible without requiring RN badge access. If a patient’s chart was flagged as “interpreter needed,” the unit clerk was responsible for placing an interpreter tablet in the patient’s room at admission, where it would remain for the entirety of the admission. The bedside nurse informed the patient and/or family that this tablet was available for medical interpretation 24/7 and instructed them on how to use it to initiate a conversation with any member of the healthcare team. We created a laminated pictorial step-by-step guide for families and providers and attached it to each interpreter tablet. When additional funding became available, a step-by-step guide with written instructions was translated into our 5 most common languages: Spanish, Somali, Nepali, Arabic, and Haitian Creole. The unit clerk was tasked with assessing the condition of the tablets and their chargers, ensuring they were functional once per 12-hour shift and performing or delegating appropriate upkeep.

### Education on Interpreter Services

Lead QI members sent emails in November 2024 to all Heart Center healthcare providers detailing the new standards for interpreter tablets. Emails were also sent to nurses and unit clerks outlining their responsibilities. In January 2025, lead QI team members guided nurses and providers through the new process for using the interpreter tablet. Champion nurses and providers were responsible for giving additional just-in-time education to nurses. The hospital’s director of language services notified the institution’s medical interpreters that parents would be allowed to call an interpreter themselves (obviating the need to wait for the bedside nurse to start the interpreter application, especially if needed urgently) via the tablet.

In March 2025, QI members conducted in-service education for interpreters about cardiac-specific anomalies and procedures to help them understand the complexity of our patient population and the importance of communication. In June 2025, QI members designed a visual cue (a heart with a puzzle and the word “interpreter”) to remind the healthcare team which patients needed interpreters. They placed it in the providers’ conference workspace for review before rounds. Starting in December 2025, the QI team also designed a translated handout to inform patients and families of their right to free interpreter services. Starting in January 2026, the healthcare team received an informational Badge Buddy regarding interpreter services.

Additionally, the QI team crafted simulation scenarios based on patient stories to mimic the experience of those who speak an LOE when interpreters are not used for critical communications. A team of Spanish-bilingual providers and native English speakers enacted scenarios with a “flipped script,” in which the bilingual providers cared for the native English speakers only in Spanish. The simulations were professionally recorded by our audiovisual production team and then converted into educational modules, which were made accessible to all hospital employees in October 2025.

### Health Literacy-Appropriate Documents

The QI team designed new health literacy-appropriate documents for patients and families to communicate the new interpreter-use process, encourage participation during FCR, and instruct parents on how to activate a rapid response if they have concerns about their child. This intervention was labor-intensive, requiring the de novo creation of the documents, revisions by the institution’s health literacy and marketing departments, and translation into Spanish, Somali, Nepali, Arabic, and Haitian Creole. This series of 15 handouts replaced the old “welcome packet” that families received at the time of admission to either unit, and it took effect in December 2025.

### Methods of Evaluation and Analysis

Our QI team received monthly reports generated by the director of language services that recorded the number of times and the duration an interpreter (in-person, by telephone, or via a tablet application) was used per patient. We used a statistical process control (SPC) u-chart to track our primary outcome metric—the monthly number of interpreter occurrences per 100 LOE-patient-days. We used an x-bar chart to track the average interpreter minutes per patient per month per 100 LOE-patient-days as our secondary outcome. To track our third outcome, the percentage of interpreter use during an emergency (cardiac arrest or rapid response), we used a p-chart, with the number of patients for whom an interpreter was used during an emergency as the numerator, and the total number of LOE patients who experienced an emergency with parents at bedside as the denominator. For the primary outcome, July 2023 to August 2024 served as the baseline data period, whereas for the secondary and tertiary outcomes, January 2022 to August 2024 served as the baseline data period. The SPC chart centerlines were shifted after special cause variation was identified using the Shewhart rules.^25^ Finally, a survey was administered to parents identified as speaking an LOE to assess their feedback.

### Process Measures

First, we tracked the percentage of parents who received education on using the interpreter tablet during their hospitalization. We assessed this monthly via completed parental surveys from December 2024 to April 2026. Second, we recorded provider chart documentation of interpreter utilization during an emergency from March 2022 to May 2026. Third, we audited the placement of the interpreter tablets in the patient’s room, ensuring that the tablets had chargers and necessary software application updates along with a magnet interpreter reminder sign outside the patient’s room. We audited this performance monthly from June 2025 to May 2026. For each process measure, control charts were developed to track progress.

### Balancing Measures

The average monthly ACCU-CTICU LOS was calculated for all patients. This was evaluated to ensure that efforts to increase interpreter use did not inadvertently lead to longer hospital stays. Additionally, the duration of morning FCR with interpreter use was audited in ACCU. This measure was necessary to ensure that our efforts to increase interpreter use did not prolong rounds.

### Ethical Considerations

Our institutional review board determined that this project met the criteria for QI work and not human subjects research and was thus institutional review board-exempt. This QI manuscript was prepared using SQUIRE 2.0 guidelines.^26^

## RESULTS

### Primary Outcome Measures

The primary outcome measure was the occurrence of interpreter use in the ACCU and CTICU (Fig. 2). The SPC u-chart demonstrates a centerline shift during the intervention period from May 2025 to June 2025: an increase from 94 to 212 occurrences per 100 LOE-patient-days, which was sustained through May 2026. The points outside the control limits reflect small numerators/denominators for November–December 2026 and large numerators/denominators for July 2025 and March 2026. Notably, those 4 points of special cause after the centerline shift suggest some system instability but did not require any adjustment to the centerline average.

**Fig. 2.**
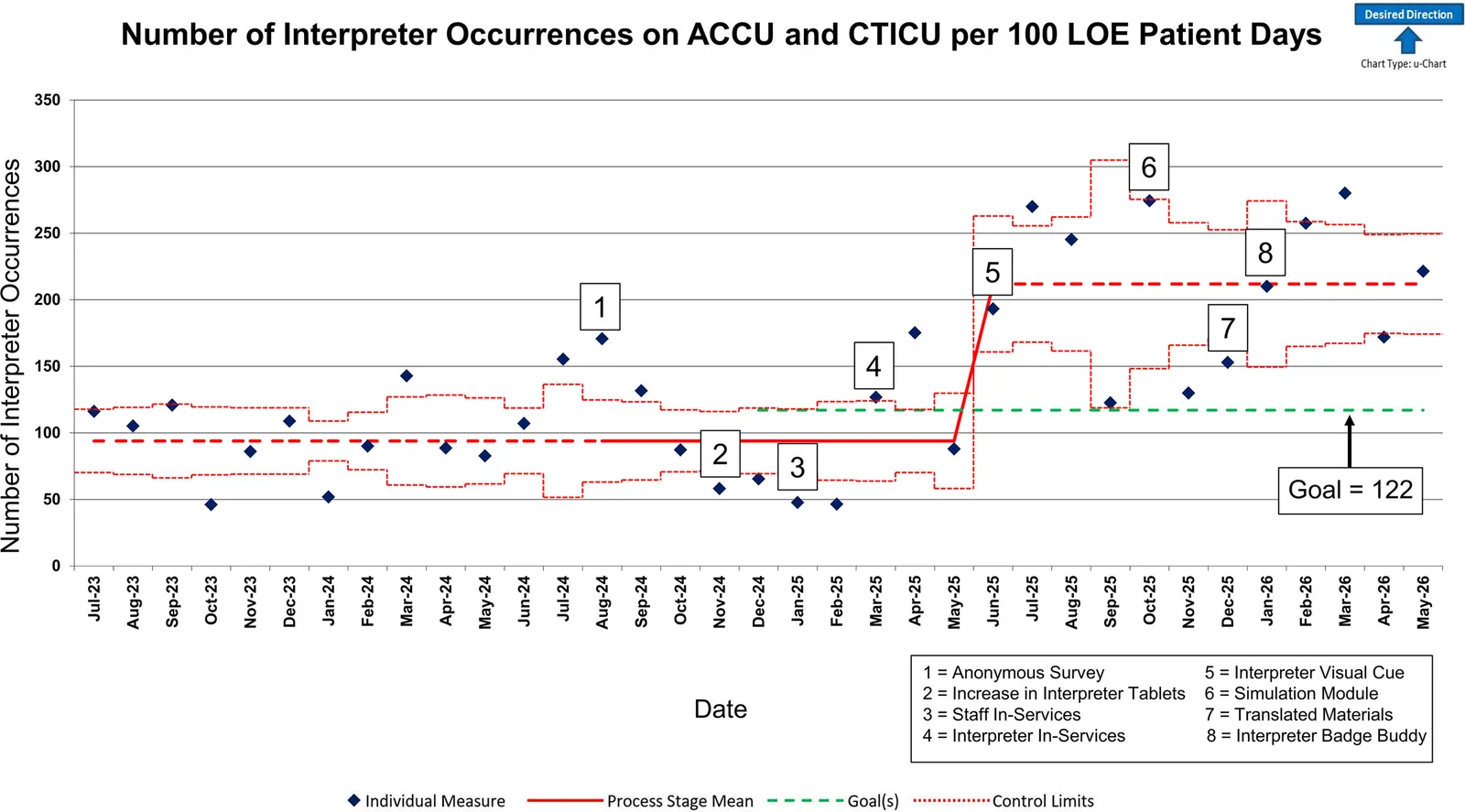
Statistical process control chart with primary outcome measure: the number of interpreter occurrences in the ACCU and CTICU per 100 patient days for LOE patients. Numbered boxes represent key interventions in order of implementation.

The secondary outcome measure was the average interpreter minutes per patient per month per 100 LOE-patient-days (Fig. 3). The SPC x-bar chart demonstrates a desired upward shift in the centerline during the intervention period from May 2025 to June 2025: an increase from 10 to 23 minutes after the interventions, which was sustained through May 2026.

**Fig. 3.**
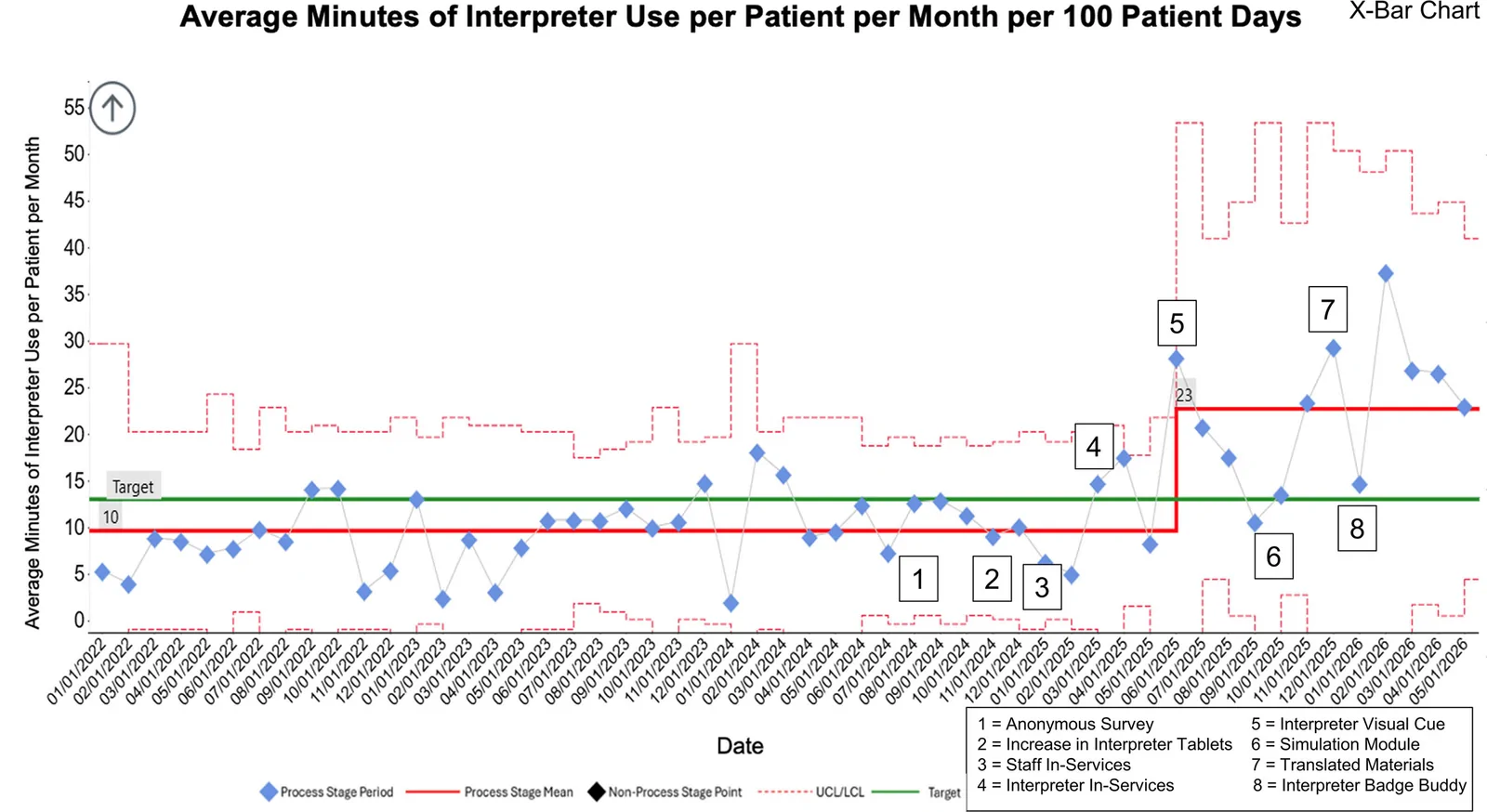
Statistical process control chart with secondary outcome measure: the average interpreter minutes per patient per month per 100 patient days for LOE patients in the ACCU and CTICU. Numbered boxes represent key interventions in order of implementation.

The third outcome measure was the percentage of LOE patients for which an interpreter was used during emergency responses (Fig. 4). The SPC p-chart displays a centerline shift in conjunction with our interventions: an increase in mean performance from 29% to 91%, which has again been sustained through May 2026. The wide control limits reflect a smaller “n” for emergent situations.

**Fig. 4.**
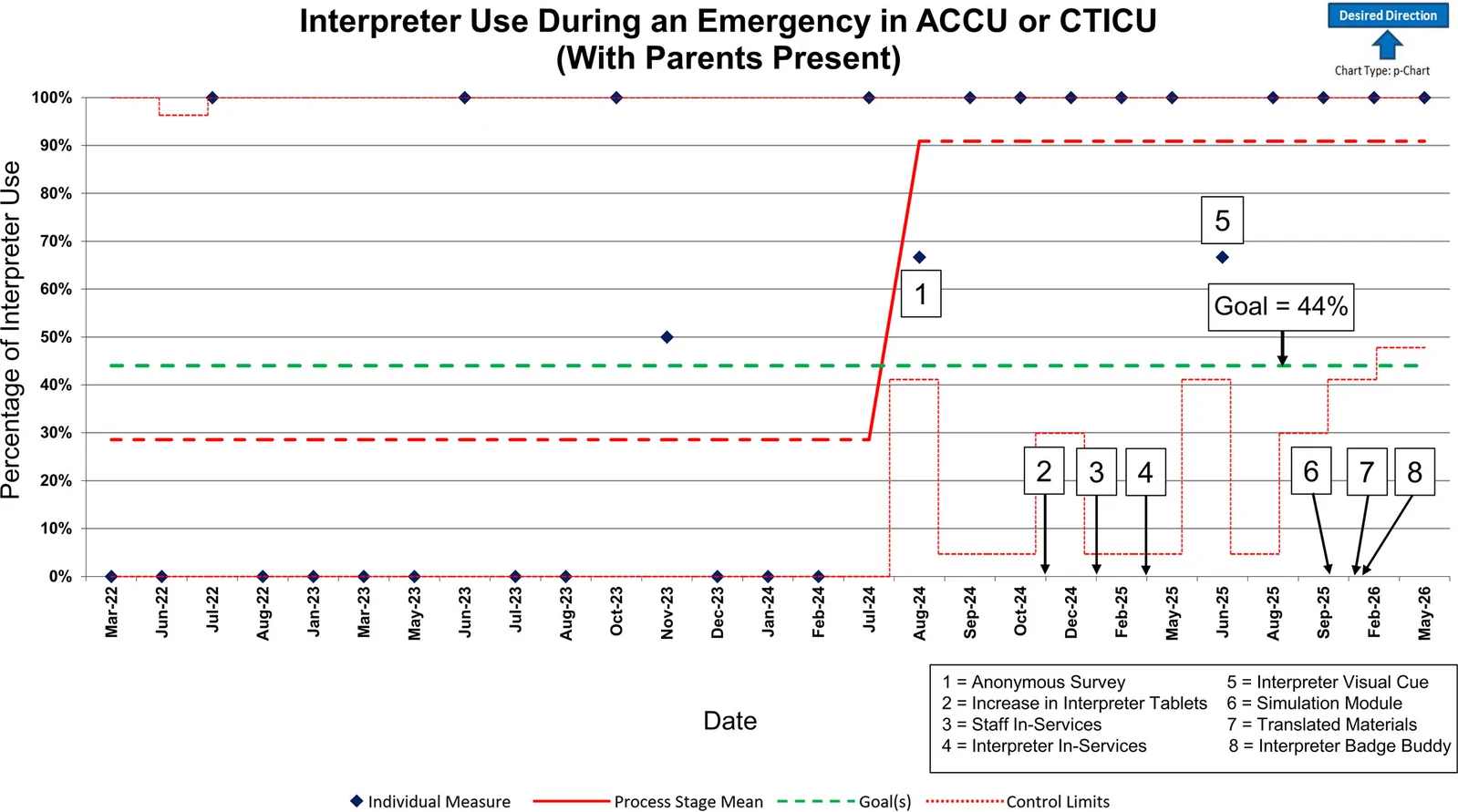
Statistical process control chart with third outcome measure: interpreter use during an emergency in the ACCU or CTICU with parents present. Numbered boxes represent key interventions in order of implementation.

### Process Measures

Parental surveys tracking the percentage of parents who received education regarding interpreter tablet use increased from 76% to 100% (see **Figure 2, Supplemental Digital Content 2,** https://links.lww.com/PQ9/A826). Monthly provider chart documentation of interpreter utilization during an emergency with parents present increased from 28% to 94% (Fig. 5). Finally, the monthly audits of placement of interpreter tablets in the patient’s room, with a magnet sign outside the patient’s room, increased from 78% to 100% (see **Figure 3, Supplemental Digital Content 3,** https://links.lww.com/PQ9/A827).

**Fig. 5.**
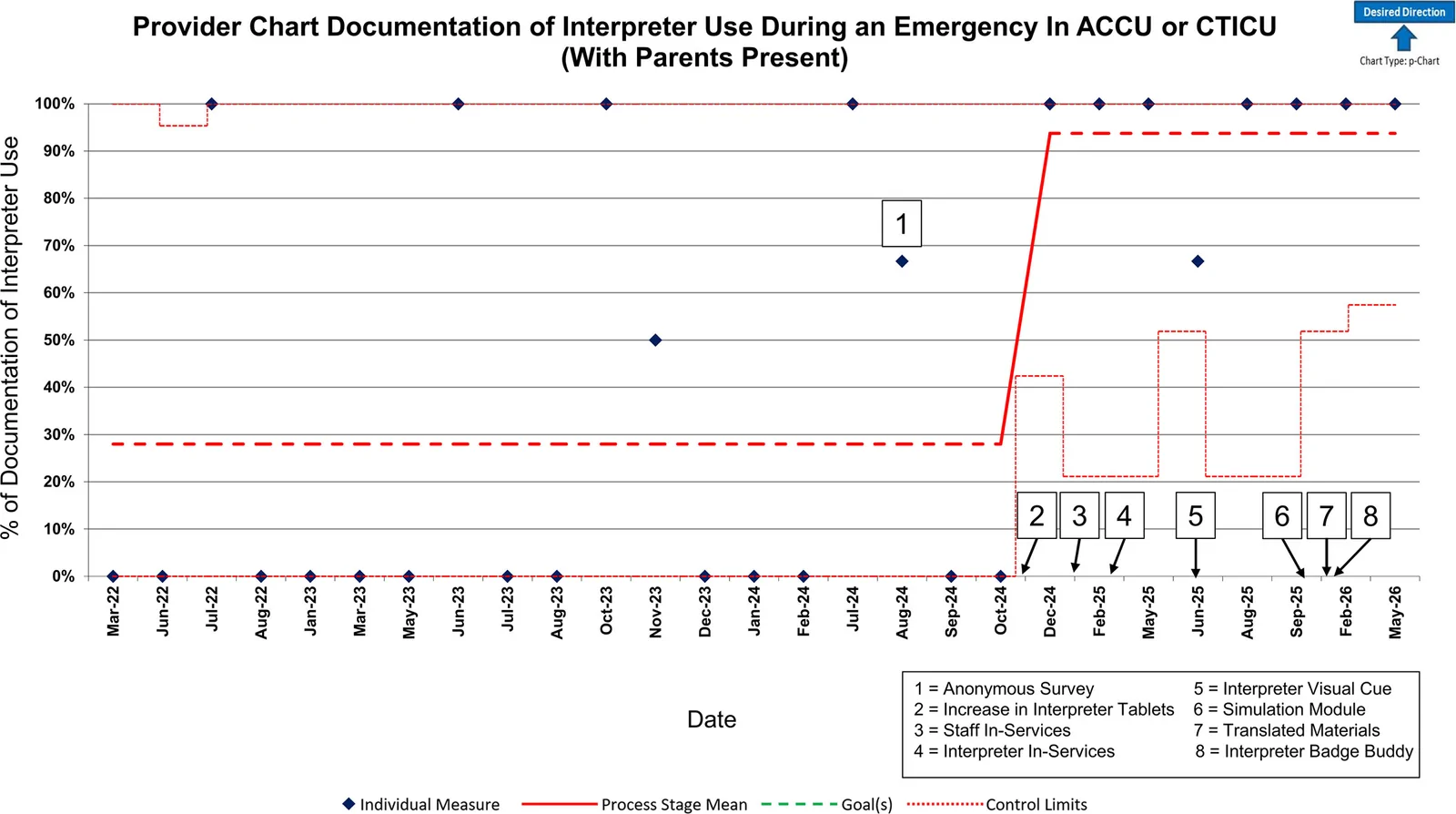
Statistical process control chart with process measure: provider chart documentation of interpreter use during an emergency in the ACCU and CTICU with parents present. Numbered boxes represent key interventions in order of implementation.

### Balancing Measures

The baseline average combined LOS was 11 days, and we observed no increase in LOS despite increased interpreter utilization (see **Figure 4, Supplemental Digital Content 4,** https://links.lww.com/PQ9/A828). The baseline average duration of FCR on the ACCU was 154 minutes, and we observed no increase in duration when interpreters were required (Fig. 6).

**Fig. 6.**
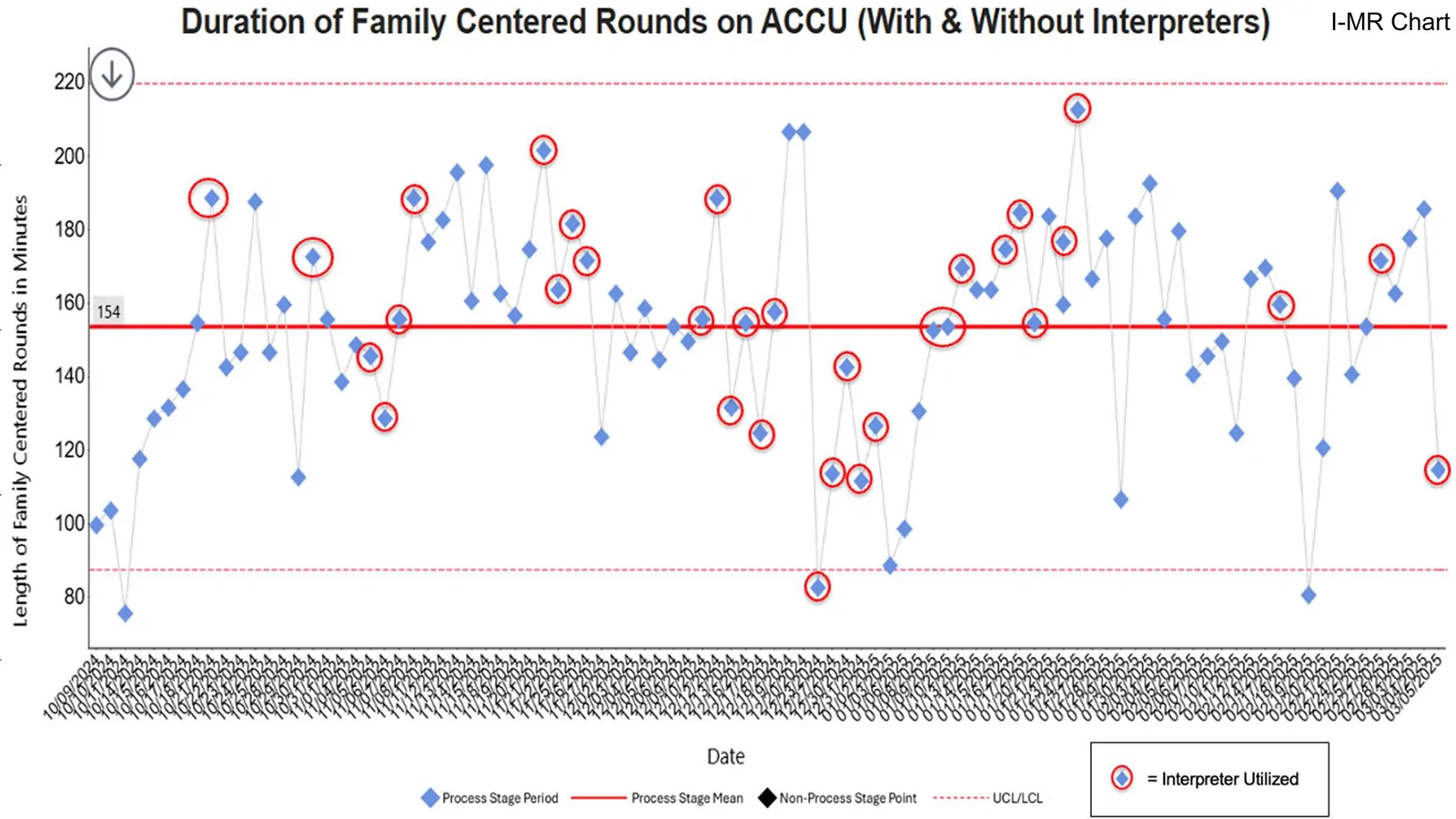
Statistical process control chart with balancing measure: the average duration of morning FCRs with interpreters in the ACCU. The points with red circles represent rounds for which parents also needed an interpreter and interpreters were utilized.

### Provider Feedback

To evaluate the impact of these new processes, multidisciplinary healthcare providers completed anonymous surveys before and after the interventions were implemented. Initially, healthcare providers reported poor access to interpreters, cumbersome processes for using them, and difficulties scheduling in-person interpreters as the primary barriers to utilizing them. Postinterventions, the results indicated a shift: healthcare providers no longer reported accessibility issues as a significant concern, and the process to activate the system became less time-intensive. Additionally, healthcare providers initially reported using an interpreter only 0–1 time/d to communicate medical updates to families, which increased to 1–3 times/d postinterventions.

### Family Engagement and Feedback

The new process received positive feedback from a linguistically diverse group of 82 parents who spoke an LOE. There were 38 Spanish-, 11 Haitian Creole-, 10 Somali-, 8 Arabic-, 4 Nepali-, 3 French-, 2 Telugu-, and 6 other (Oromo, Turkish, Dari, Tigrinya, Pashto, or Lingala) speaking respondents. The feedback focused on the utility of the new process and their satisfaction. Fifty-five percent of parents operated the interpreter tablet themselves and reported they felt comfortable doing so. Open-ended feedback revealed that 24/7 access to interpretation fostered gratitude, empowerment, and inclusion in their child’s care, and all 82 respondents recommended continuing the service for all families.

## DISCUSSION

Given the underutilization of interpreters nationally^12–14^ and at our center, our QI team focused on the role healthcare institutions have in optimizing interpreter infrastructure and accessibility.^27^ Our QI team implemented interventions to increase interpreter utilization, addressing a significant opportunity for improvement. Using QI methodology, we successfully increased interpreter use among hospitalized families who speak an LOE, achieving this aim without a significant increase in hospital LOS or FCR time (balancing measures). Our primary and secondary outcomes SPC charts show a centerline shift in our data in June 2025, 7 months after the increase in the number of available interpreter tablets on the units. Expanding and standardizing the availability of interpreter tablets made it easier for healthcare providers to use, resulting in increased interpreter use. This shift reflected not only an increase in interpreter use and the duration of communication with LOE families, but also a positive change in healthcare providers’ survey responses. Key interventions that directly increased interpreter use included targeted team engagement and educational initiatives, which ensured healthcare providers were well-informed about the new standard of care.

We also successfully increased the percentage of providers documenting interpreter use for LOE patients in the ACCU and CTICU during rapid or emergency responses. The cumulative effect of our interventions increased interpreter accessibility and raised awareness of interpreter use among healthcare providers. Based on survey responses, these interventions also empowered parents to freely use an available interpreter tablet to share real-time patient concerns with healthcare providers and actively engage in patient decision-making during these emergent situations. Similar interventions to increase interpreter use in different healthcare settings, such as the pediatric intensive care unit^18^ and emergency department, have been reported^19–21^ and offer effective strategies that can be successfully implemented across a variety of clinical areas to optimize interpreter utilization. However, our efforts to empower families to initiate interpreter services at any given time during their hospital stay were successful and fill a gap that has not been seen in other studies.^18^

We intended to standardize communication between healthcare providers and patients and families so that they feel part of the team throughout their hospitalization. Similar barriers to using interpreters in healthcare settings, such as limited interpreter availability and a time-intensive process, have been identified previously and align with our findings.^4,17^ Prior studies also demonstrate that raising awareness and knowledge of the importance of interpreter use through education^17^ and simulation^27^ were effective at reducing barriers to adequate interpreter use.^17,27^ As evidenced by our postintervention healthcare provider survey, the increased self-reported use of interpreters aligns with our data showing increased use of interpreter services over the course of the project. Further research is needed to understand the effect of increased interpreter duration; however, we hypothesize that it would lead to more detailed and comprehensive discussions between the families and the healthcare team.

The findings from this QI project should be interpreted in the context of several limitations. This study was limited to a single-center institution that has a wide variety of languages and interpreter services. Furthermore, our institution has resources such as a language service department with both interpretation and translation capabilities, as well as access to interpreter tablets. Other institutions may lack these language resources and thus may have difficulty replicating our work. Finally, our institution is deeply committed to QI work and provides workforce and capital support for QI initiatives. This degree of support may not be available at other institutions.

## CONCLUSIONS

Standardizing and improving access to interpreter services can increase the use of interpreters among ACCU and CTICU patients and families. The most effective interventions are those that make interpreters more accessible to both healthcare teams and families. Encouraging families to initiate interpreter services independently enhances communication and fosters a greater sense of belonging. As an institution that highly values improving communication and helping families feel welcomed, this project successfully cultivated our institution’s core values. Through enhanced communication and family engagement, healthcare providers can improve families’ understanding of their child’s medical state and anticipatory needs. Future work will evaluate whether the use of standardized interpreters improves patient outcomes, including emergency department visits and hospital readmissions.

## ACKNOWLEDGMENTS

The authors wish to acknowledge and thank the team members who were active participants in this QI project: Avery Adams, Julie Aldrich, Antonio Cabrera, Lavone Caldwell, Tess Coakley, Tina Cole, Christina Conkel, Madison Cooke, Valerie Huang, Meredith Lough, Sara Marsalek, Nibitanga Melanie, Jennifer Ochall, Arash Salavitabar, Brenna Sheeley, Wendy Stanzione, Cristina Tomatis Souverbielle, Ahquilah Tucker, Roxann Tyner, and Taise Young. The authors thank Andrew Bethune for his review of this manuscript. Artificial intelligence, ChatGPT, was used to help shorten the introduction and concluding paragraphs.

## Supplementary Material


