# Supplementary material for: Increasing Interpreter Use for Pediatric Cardiology Units: A Quality Improvement Initiative
Source: Pediatr Qual Saf. 2026 Sep 28;11(5):e933. doi: 10.1097/pq9.0000000000000933 (PMC13619301; doi:10.1097/pq9.0000000000000933)

Process Map for Patients Admitted to ACCU and CTICU, Rapid Response and Code Blues

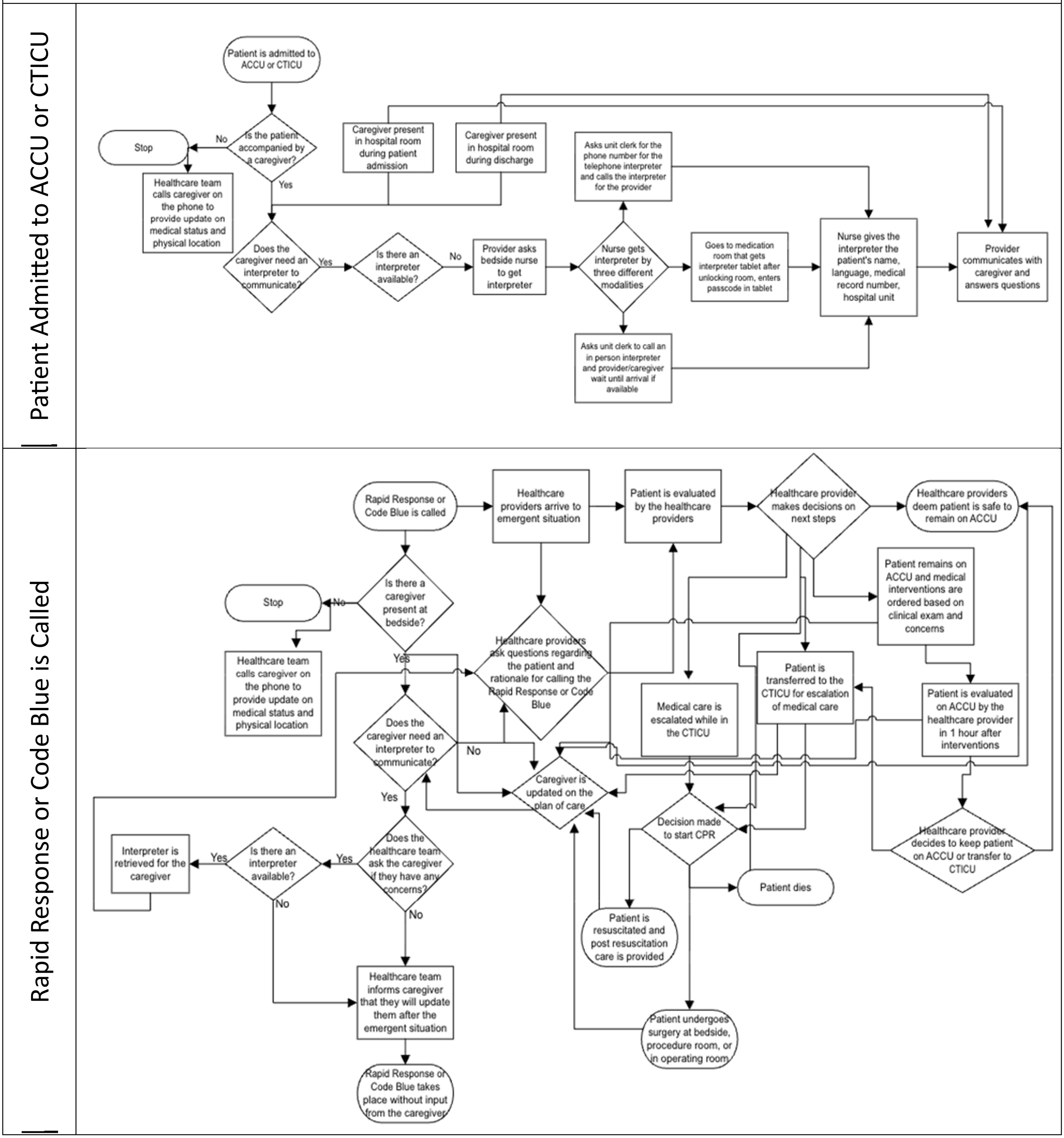

Supplement: Supplementary file 1 [file pqs-11-e933-s001.pdf]
