# Supplementary material for: Increasing Interpreter Use for Pediatric Cardiology Units: A Quality Improvement Initiative
Source: Pediatr Qual Saf. 2026 Sep 28;11(5):e933. doi: 10.1097/pq9.0000000000000933 (PMC13619301; doi:10.1097/pq9.0000000000000933)
Supplement: Supplementary file 2 [file pqs-11-e933-s002.pdf]

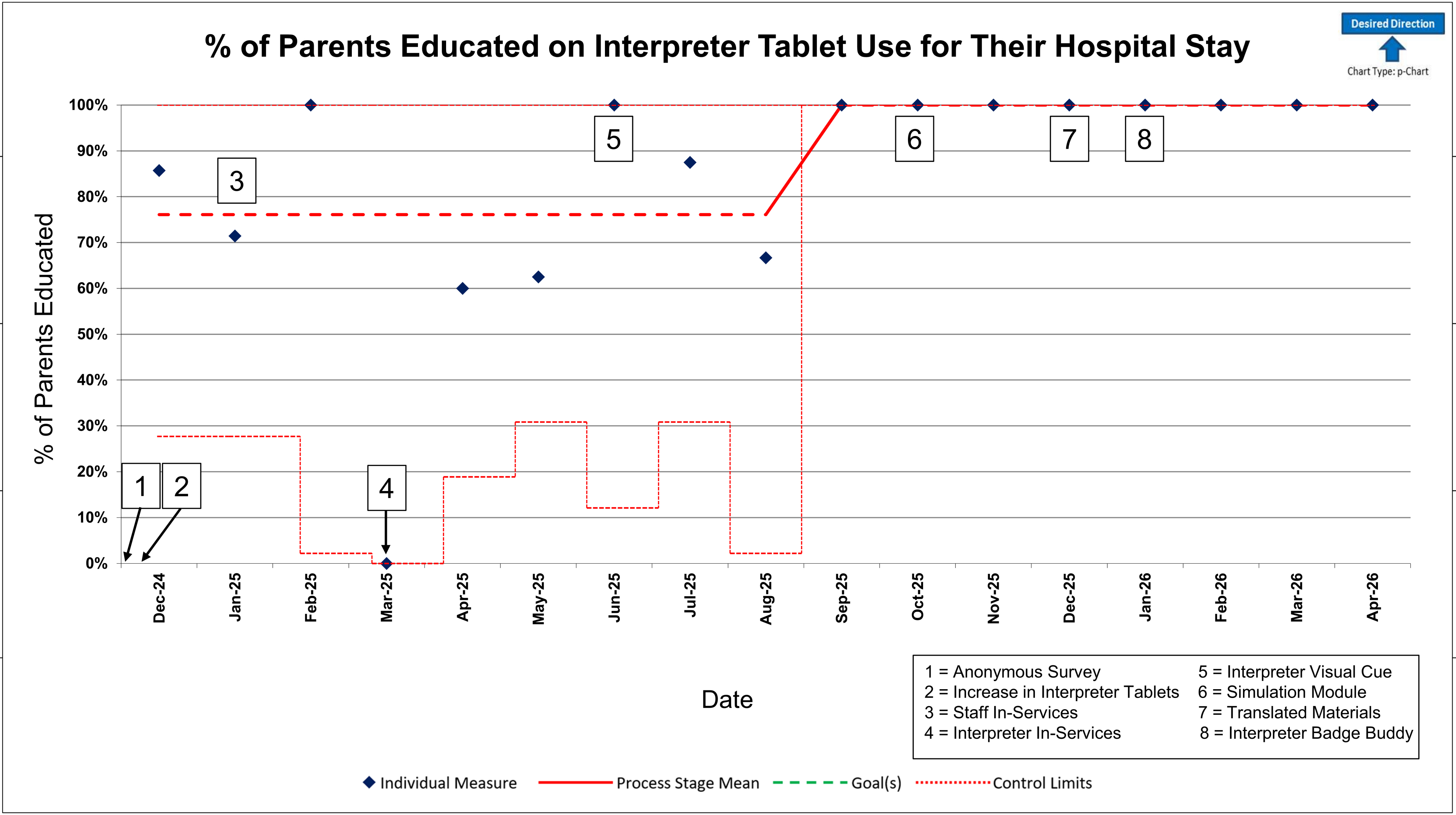

% of Parents Educated on Interpreter Tablet Use for Their Hospital Stay
Desired Direction
Chart Type: p-Chart
% of Parents Educated
100%
90%
80%
70%
60%
50%
40%
30%
20%
10%
0%
1
2
3
4
5
6
7
8
Dec-24
Jan-25
Feb-25
Mar-25
Apr-25
May-25
Jun-25
Jul-25
Aug-25
Sep-25
Oct-25
Nov-25
Dec-25
Jan-26
Feb-26
Mar-26
Apr-26
Date
1 = Anonymous Survey
2 = Increase in Interpreter Tablets
3 = Staff In-Services
4 = Interpreter In-Services
5 = Interpreter Visual Cue
6 = Simulation Module
7 = Translated Materials
8 = Interpreter Badge Buddy
Individual Measure
Process Stage Mean
Goal(s)
Control Limits
