# Supplementary material for: Increasing Interpreter Use for Pediatric Cardiology Units: A Quality Improvement Initiative
Source: Pediatr Qual Saf. 2026 Sep 28;11(5):e933. doi: 10.1097/pq9.0000000000000933 (PMC13619301; doi:10.1097/pq9.0000000000000933)
Supplement: Supplementary file 4 [file pqs-11-e933-s004.pdf]

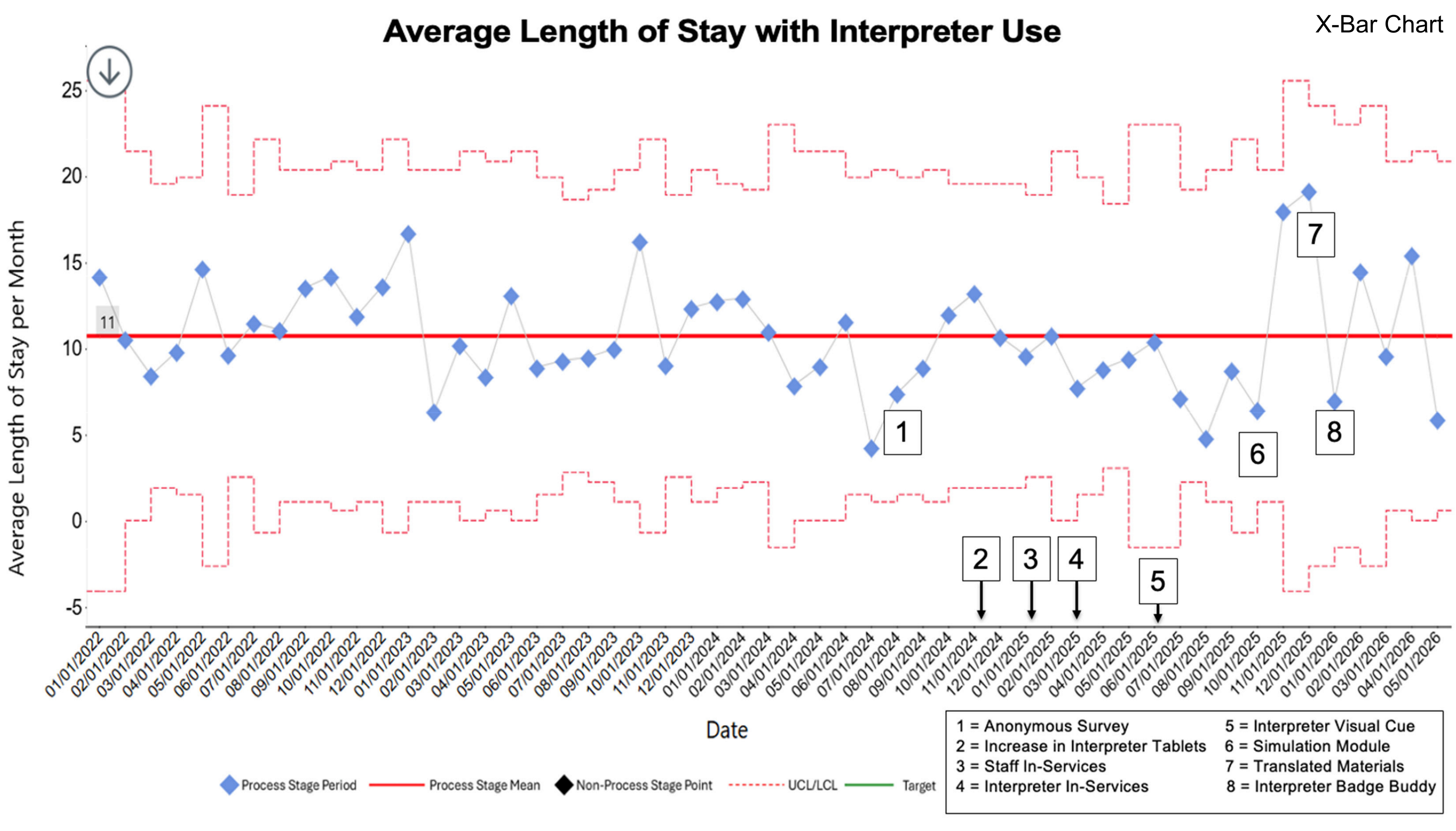

Average Length of Stay with Interpreter Use
X-Bar Chart
Average Length of Stay per Month
Date
11
1
2
3
4
5
6
7
8
Process Stage Period
Process Stage Mean
Non-Process Stage Point
UCL/LCL
Target
1 = Anonymous Survey
2 = Increase in Interpreter Tablets
3 = Staff In-Services
4 = Interpreter In-Services
5 = Interpreter Visual Cue
6 = Simulation Module
7 = Translated Materials
8 = Interpreter Badge Buddy
